# Inhibition of SARS-CoV-2 by Targeting Conserved Viral RNA Structures and Sequences

**DOI:** 10.3389/fchem.2021.802766

**Published:** 2021-12-23

**Authors:** Shalakha Hegde, Zhichao Tang, Junxing Zhao, Jingxin Wang

**Affiliations:** Department of Medicinal Chemistry, University of Kansas, Lawrence, KS, United States

**Keywords:** SARS-CoV-2, antiviral, RNA-targeting, small molecule, antisense oligonucleotide, untranslated region, programmed frameshift, RIBOTAC

## Abstract

The ongoing COVID-19/Severe Acute Respiratory Syndrome CoV-2 (SARS-CoV-2) pandemic has become a significant threat to public health and has hugely impacted societies globally. Targeting conserved SARS-CoV-2 RNA structures and sequences essential for viral genome translation is a novel approach to inhibit viral infection and progression. This new pharmacological modality compasses two classes of RNA-targeting molecules: 1) synthetic small molecules that recognize secondary or tertiary RNA structures and 2) antisense oligonucleotides (ASOs) that recognize the RNA primary sequence. These molecules can also serve as a “bait” fragment in RNA degrading chimeras to eliminate the viral RNA genome. This new type of chimeric RNA degrader is recently named ribonuclease targeting chimera or RIBOTAC. This review paper summarizes the sequence conservation in SARS-CoV-2 and the current development of RNA-targeting molecules to combat this virus. These RNA-binding molecules will also serve as an emerging class of antiviral drug candidates that might pivot to address future viral outbreaks.

## Introduction

### SARS-CoV-2’s Life Cycle and “Druggable” Targets

**FIGURE 1:**
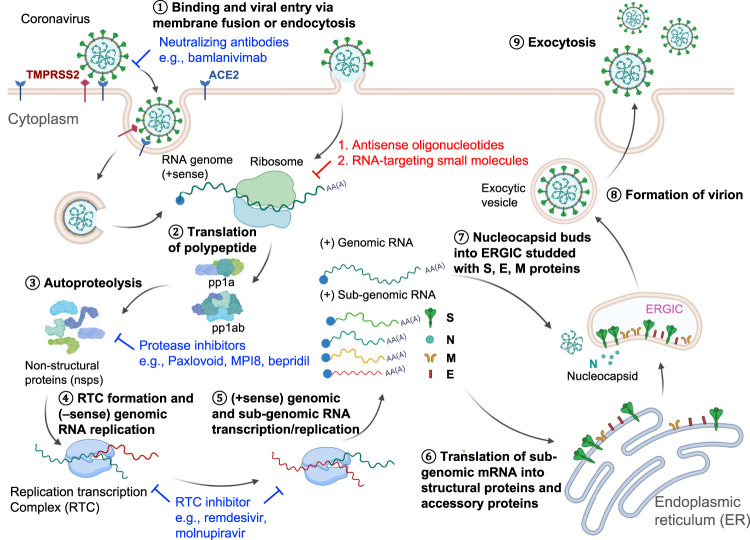
SARS-CoV-2 Life cycle and viral targets for antiviral development. ① The virus enters the host cell through endocytosis using spike protein-angiotensin-converting enzyme 2 (ACE2) interaction. ② The host ribosome then translates the positive-sense RNA genome. ③ The long polypeptide precursor is subsequently cleaved by the viral proteases into non-structural proteins (nsp), which will assemble the replication-transcription complex (RTC) for ④ viral RNA genome replication in the 3’→5’ direction and ⑤ transcription in the 5’→3’ direction for the whole genome and sub-genomic sequences. ⑥ The host ribosome further translates the sub-genomic sequences that encode the nucleocapsid proteins. ⑦–⑧ Newly synthesized nucleocapsid components are assembled in the endoplasmic reticulum-Golgi intermediate compartment (ERGIC) to form the infectious virions, which are ⑨ released from the cell by maturation in the budding process. Some anti-SARS-CoV-2 agents illustrated in this figure include spike protein neutralizing antibody bamlanivimab, main protease inhibitors PF-07321332, MPI8, and bepridil, and RdRP inhibitors remdesivir and molnupiravir.

SARS-CoV-2 belongs to the betacoronavirus genus and is an enveloped ssRNA (+) virus with a genome length of about 30,000 nucleotides (RefSeq NC_045512) (Wu et al., 2020). The viral genome is 5’ capped and 3’ polyadenylated (Robson et al., 2020) so that it is recognized and treated as an mRNA by the host cell ribosome. Two-thirds of the viral genome at the 5’-end have two long open reading frames (ORFs), ORF1a and ORF1ab, encoding two replicase-associated polyprotein precursors, pp1a and pp1ab (Figure 1). These polyprotein precursors are cleaved by viral proteases into 16 non-structural proteins (nsps) (Kim et al., 2020), some of which have essential viral functions (Figure 1). For example, an RNA-dependent RNA polymerase (RdRP) complex consisting of nsp12 in pp1ab and nsps7 and 8 in pp1a is required for viral transcription and replication (Hillen et al., 2020). RdRP is the core enzyme in the viral “replication-transcription complex” (RTC) (Fung and Liu, 2019). The RTC then promotes 3’→5’ replication of the (–) viral genome to form a full-length double-stranded (ds) RNA in the endoplasmic reticulum (ER) membrane invaginations (Knoops et al., 2008). This dsRNA then serves as a template for transcribing the genomic and subgenomic RNAs by RTC-mediated transcription in the 5’→3’ direction (Wu and Brian, 2010) (Figure 1). RNA transcription for each coronavirus structural protein is accomplished through a “discontinuous” mechanism. The RTC binds to the leader transcriptional regulatory sequences (TRS-L) in the 5’ UTR and then “hops” onto the body TRS (TRS-B) sequence. These TRS-B sequences locate at the 5’-end of each structural gene for transcription (Zúñiga et al., 2004; Sola et al., 2015). After completing structural protein synthesis and genomic RNA replication, new coronavirus particles are assembled at the host ER and released through the Golgi apparatus to complete the viral life cycle (Sawicki et al., 2007).

Current drug development pipelines have tackled different steps in the life cycle of SARS-CoV-2 (Figure 1). Spike protein-targeting antibodies (e.g., bamlanivimab) can effectively neutralize the virus and prevent viral entry (Gottlieb et al., 2021). RNA-targeting antisense oligonucleotides (ASO) or small molecules will degrade the viral RNA genome or hinder RNA translation (Li et al., 2021b, Li et al., 2021a.; Lulla et al., 2021; Rosenke et al., 2021; Sun et al., 2021a; Zhang et al., 2021; Zhu et al., 2021). The SARS-CoV-2 main protease (M^pro^) is also an attractive drug target. PF-07321332 (Paxlovoid) was developed as an oral drug targeting M^pro^ and is being tested in a Phase 3 clinical trial (ClinicalTrials.gov Identifier: NCT04960202) (Owen et al., 2021). Other reported M^pro^ inhibitors such as an FDA-approved drug, bepridil, and a peptoid MPI8 were demonstrated to have efficacy in virus-infected cells (Ma et al., 2021; Vatansever et al., 2021). RdRP inhibitors remdesivir and molnupiravir, which impede the RNA replication/transcription processes, both showed clinical improvement in the COVID-19 patients (Wang et al., 2020; Fischer et al., 2021). In this review, we focused on the RNA-targeting approach, an emerging antiviral pharmacological modality that is complementary to traditional protein-targeting methods. An advantage of ASO-based drug development is the ability to rapidly generate drug candidates, which recognize the primary sequences of viral RNAs. The off-targets of the ASOs can also be quickly identified through experiments or predictive algorithms based on the primary sequences (Hagedorn et al., 2018; Yoshida et al., 2019). Compared to the ASO-based drug discovery, RNA-targeting small molecules are a relatively underdeveloped field. To date, only one non-ribosomal RNA binding molecule, risdiplam, has been approved by the FDA (Jaklevic, 2020). We envision that the chemical space, potency, off-targets for RNA-binding small molecules will be further investigated as therapeutics to antivirals and other human diseases (Hargrove, 2020; Meyer et al., 2020; Ursu et al., 2020). RNA-targeting molecules will probably synergically inhibit viral replication when combined with protein-targeting drugs in cocktail therapies.

### Conserved RNA Sequences and Structures in SARS-CoV-2

**FIGURE 2:**
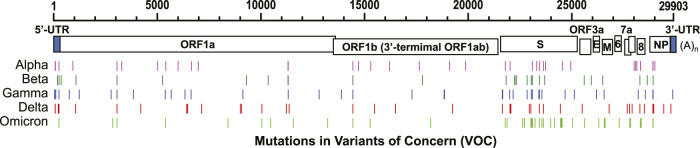
Nucleotide mutations in five VOCs compared to the original SARS-CoV-2 sequence discovered in Wuhan, China (RefSeq NC_045512). GenBank accession numbers: B.1.1.7 (Alpha): MZ344997, B.1.351 (Beta): MW598419, P.1 (Gamma): MZ169911, and B.1.617.2 (Delta): MZ359841. GISAID accession number: B.1.1.529 (Omicron): EPI_ISL_6795188.

**FIGURE 3:**
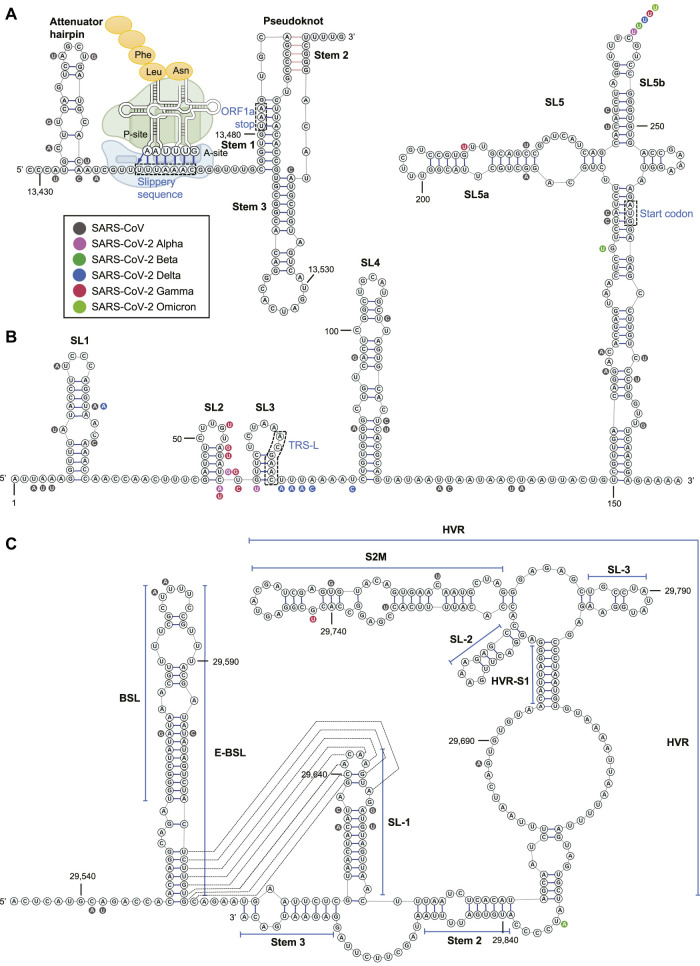
The RNA structure and nucleotide conservation of the **(A)** PFS element in SARS-CoV-2, **(B)** 5’ UTR, and **(C)** 3’ UTR. The ribosome acts on the slippery sequence to produce a –1 PFS is illustrated in **(A)**.

The mutation rate of SARS-CoV-2 is estimated at 1 × 10^−3^ substitutions per base (30 nucleotides/genome) per year under neutral genetic drift conditions (van Dorp et al., 2020), or 1 × 10^−5^–1×10^−4^ substitutions per base (0.3–3 nucleotides/genome) in each transmission events from population phylodynamic studies (Van Egeren et al., 2021). This rate is much slower than some other RNA viruses, such as influenza A virus (Manzanares-Meza and Medina-Contreras, 2020) and human immunodeficiency virus (HIV) (Van Egeren et al., 2021). As of December 2, 2021, five circulating variants of SARS-CoV-2 are classified as variants of concern (VOC) in the World Health Organization (https://www.who.int/en/activities/tracking-SARS-CoV-2-variants/), including B.1.1.7 (Alpha), B.1.351 (Beta), P.1 (Gamma), B.1.617.2 (Delta), and B.1.1.529 (Omicron) variants. Among these variants, the spike protein (S) harbors most of the nucleotide mutations compared to the original genomic sequence isolated from Wuhan, China, in December 2019 (Figure 2). Some mutations occur beyond the protein-coding region. For example, a prevalent mutation C241U (c.–63C>U) exits in the 5’ untranslated region (UTR) of all four VOCs (Figure 3A).

By phylogenetic comparison of SARS-CoV-2, SARS-CoV, and SARS-related bat coronavirus sequences (Ceraolo and Giorgi, 2020), an earlier bioinformatics work from the Das group in 2020 identified 30 RNA regions as SARS-related conserved sequences and predicted 106 regions as SARS-CoV-2 conserved structures (Rangan et al., 2020). Shortly afterward, the RNA structures of SARS-CoV-2 were interrogated by chemical probing (Lan et al., 2020; Manfredonia et al., 2020; Sanders et al., 2020; Zhao et al., 2020; Sun et al., 2021a; Huston et al., 2021), psoralen crosslinking (Ziv et al., 2020), and NMR spectroscopy experiments (Wacker et al., 2020). Among all SARS-CoV-2 RNA structures identified, the 5’ and 3’ UTRs and a region named programmed –1 frameshift (PFS) element (13,459–13,546) in the ORF1a/ab have been intensively studied for their structures, functions, and druggability.

#### SARS-CoV-2 PFS Element

ORF1a is the 5’-terminal fraction of ORF1ab and has an in-frame stop codon at nucleotide 13,481. The correct translation of ORF1b (3’-terminal ORF1ab), which encodes the viral RdRP (nsp12), requires a PFS that shifts the ORF by –1 nucleotide via a “slippery sequence” to circumvent the ORF1a stop codon (Hagemeijer et al., 2012) (Figure 3A). Although the PFS element was not shown as a conserved structure in Das’ bioinformatics algorithm (Rangan et al., 2020), this region has demonstrated high-degree conservation among SARS-CoV and four VOC of CoV-2 (Figure 3A). The PFS element contains an attenuator hairpin (a negative regulator of the PFS), a slippery sequence (U_UUA_AAC motif), and a pseudoknot structure in betacoronavirus (Hagemeijer et al., 2012; Rangan et al., 2020) (Figure 3A). Once the ribosome recognizes the pseudoknotted structure, tRNAs in the ribosomal P- and A-sites re-bind to the –1 reading frame at the slippery sequence, and the ribosome starts to translate within the new reading frame (Bhatt et al., 2021) (Figure 3A). Without PFS, viral RNA translation would halt at the stop codon (13,481–13,483) within the pseudoknot (Figure 3A). It was demonstrated that the PFS element sequence alone could recapitulate the PFS activity without a protein cofactor in SARS-CoV (Baranov et al., 2005). The pseudoknotted structure was observed in NMR (Liphardt et al., 1999), chemical probing (Huston et al., 2021), cryo-EM (complexed with an elongating ribosome) (Bhatt et al., 2021), and X-ray crystallography (Roman et al., 2021).

#### SARS-CoV-2 UTRs

In the 5’ UTR (1–265), there are five stem-loops identified, SL1–5 (Figure 3B). SL1 was demonstrated to bind to nsp1 protein and cooperate in recruiting the human ribosome (Vankadari et al., 2020). SL5, which includes the genome start codon, is a four-helix junction essential for viral packaging (Escors et al., 2003) (Figure 3C). It is proposed that the structures of SL1, SL2, and SL4, but not the exact nucleotide sequences, play a more critical role in betacoronavirus function (Yang and Leibowitz, 2015).

In the 3’ UTR, three main secondary structures were elucidated by chemical probing: bulged stem-loop (BSL), SL-1, and the highly variable region (HVR) (Figure 3C). Bioinformatics analysis and reverse genetics suggested the pseudoknotted structure formation at the base stem of BSL and the SL-1 loop in SARS-CoV (Goebel et al., 2004) (Figure 3B). The equilibrium between the double stem-loop and pseudoknot was proposed to be a molecular switch in SARS-CoV RNA transcription (Yang and Leibowitz, 2015). This equilibrium model is also supported by quantitative covariation analysis (Rfam: RF11065) (Mathews et al., 2004). However, the pseudoknot was not observed as a stable structure at 37°C in NMR experiments in a model betacoronavirus, mouse hepatitis virus (MHV) (Stammler et al., 2011). Chemical probing experiments also suggested the unfavorable formation of pseudoknot (Zhao et al., 2020; Huston et al., 2021).

The HVR in the 3' UTR is not essential to betacoronavirus. The HVR can be deleted without affecting viral propagation in cell culture, albeit the HVR-deleted MHV strain has lower pathogenicity in mice (Goebel et al., 2007). Nevertheless, some sub-region of the HVR is highly conserved among betacoronavirus, such as the stable S2M (Rangan et al., 2020) (Figure 3C). The Stem 3 region duplexed by a sequence at the 3’-end of the viral genome and that between BSL and SL-1 (Figure 3C) was shown to be essential for the MHV viability (Goebel et al., 2007; Liu et al., 2013) and phylogenetically conserved (Züst et al., 2008), although chemical probing result suggested that the formation of Stem 3 is not favorable (Zhao et al., 2020). It was demonstrated by psoralen crosslinking that the 3’-end of the genome in the Stem 3 region can bind to the viral 5’ UTR and cyclize the SARS-CoV-2 genome (Ziv et al., 2020).

Among the 106 predicted conserved structured RNA regions by the Das group (Rangan et al., 2020), two locate in the 5’ UTR: SL2–4 and SL5 (SARS-CoV-2-conserved structure-8 and -16), and one reside in the 3’ UTR: BSL-SL-1 region (SARS-CoV-2-conserved structure-33) (structure numbers provided in Rangan et al., 2020).

## Viral RNA-Targeting Strategies

### RNA-Binding Small Molecules Targeting the SARS-CoV-2 RNA Genome

De novo design of nucleic acid ligands has been pursued for more than 35 years. The field was first pioneered by the Dervan group in optimizing DNA-binding molecules (Dervan, 1986), and then by the Disney group to identify selective RNA-binding molecules. In the recent 15 years, Disney and others have established that “the right” synthetic small molecules can indeed bind to RNA structures, but not the primary sequences, with a high degree of selectivity (Fedorova et al., 2018; Warner et al., 2018; Hargrove, 2020; Ursu et al., 2020).

Viruses make use of their RNA structures to hijack host cell functions and promote viral life cycle progression. These viral RNA structures have been chosen as druggable targets in small-molecule drug development. For example, HIV-1 uses trans-activator protein (Tat) to interact with a highly structured transactivation response (TAR) hairpin in its RNA to enhance the viral transcription (Sophie et al., 1990; Schulze-Gahmen and Hurley, 2018). Peptoid inhibitors targeting the TAR-Tat interaction have been shown to inhibit HIV-1 replication *in vitro* and *in vivo* (Hamy et al., 1997).

**FIGURE 4:**
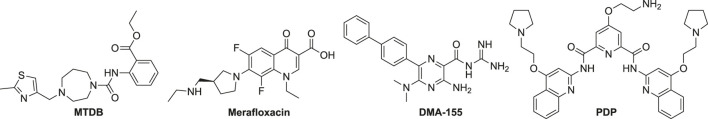
The small molecules with antiviral activities targeting the SARS-CoV-2 RNA genome.

The discovery of RNA-targeting anti-SARS-CoV or CoV-2 small molecules primarily focused on the PFS element. MTDB was first identified by virtual screening and 3-dimensional (3D) modeling. MTBD can potently bind to the pseudoknot in the SARS-CoV PFS element and inhibit the PFS function in a dual luciferase system (Park et al., 2011) (Figure 4). The dual luciferase assay is widely used in discovering and validating PFS regulators. In this assay, the PFS element was placed in the junction of a Renilla/firefly fusion luciferase, and the fusion luciferase could only be produced when the PFS occurred (Harger and Dinman, 2003). It was demonstrated by small-angle X-ray scattering analysis and reverse genetics that the conformation and function of the pseudoknot in the PFS element between SARS-CoV and SARS-CoV-2 are highly similar (Kelly et al., 2020). Indeed, MTDB can also reduce the SARS-CoV-2 PFS activity by 60% (Kelly et al., 2020).

A mCherry/GFP dual fluorescent protein assay was used in a high-content imaging screen, which identified a novel small-molecule PFS inhibitor, merafloxacin (Figure 4). Merafloxacin had a half-maximal inhibitory concentration (IC_50_) in the dual fluorescent protein reporter cells at 19 μM and SARS-CoV-2-infected cells at 2.4 μM (Sun et al., 2021b). Merafloxacin belongs to the fluoroquinolone class known to interact with bacterial DNA and gyrase/topoisomerases (Aldred et al., 2014). Merafloxacin had a similar inhibitory effect to the reporter cells with mutated PFS elements, further suggesting that merafloxacin recognizes shape but not the primary sequence of the RNA (Sun et al., 2021b). Comparing MTDB and merafloxacin side-by-side, it was demonstrated that merafloxacin was a more potent inhibitor against PFS in SARS-CoV-2-infected Vero E6 cells (Bhatt et al., 2021).

Amiloride analogs (e.g., DMA-155, Figure 4) targeting the SARS-CoV-2 5’ UTR also exhibited antiviral activity in SARS-CoV-2-infected cells (Zafferani et al., 2020). NMR studies uncovered that SL4, SL5a, and SL6 could all bind to the amilorides (Zafferani et al., 2020). An RNA sequence (RG-1) having a high propensity to form a G-quadruplex (G4) in the SARS-CoV-2 genome was validated in the coding sequence of nucleocapsid phosphoprotein (N) in cells (Zhao et al., 2021). PDP was demonstrated to stabilize RG-1 G4 and reduce the protein levels of the viral N protein by inhibiting its translation both *in vitro* and *in vivo* (Zhao et al., 2021).

Several RNA-binding proteins (RBPs) in the host cells (e.g., IGF2BP1, hnRNP A1, and TIA1) were predicted to bind to the SARS-CoV-2 RNA genome (Sun et al., 2021a). Some FDA-approved small-molecule drugs, such as nilotinib, sorafenib, and deguelin, were demonstrated to interfere with essential RBP-viral RNA interactions and reduce the viral titer (Sun et al., 2021a). Strictly speaking, the targets of these drugs are host factors rather than viral RNA structures.

### RNA-Binding ASOs Targeting the SARS-CoV-2 RNA Genome

#### Pharmacological Mechanisms of ASOs

ASOs are RNA or DNA sequences with 15–25 natural or modified nucleotides (Dhuri et al., 2020), which hybridize specifically *via* Watson-Crick base-pairing to a target RNA and modulate RNA splicing or gene expression (Roberts et al., 2020). ASOs generally act through two mechanisms in human cells: 1) cleaving of the target RNA *via* ASO-induced ribonuclease (RNase) H1 activity and 2) masking the target RNA from interaction with the human RBPs or the ribosome.

The ASOs used to induce RNase H1 activation are also termed “gapmers”. Gapmers usually contain a central DNA sequence (> 6 nucleotides) that hybridizes with the target RNA (Papargyri et al., 2020). RNase H1 is a ubiquitous ribonuclease found in the nucleus and the cytoplasm of all human cells (Crooke, 2017). RNase H1 specifically recognizes and hydrolyzes the RNA strand of the RNA-DNA heteroduplexes formed between the DNA block in the gapmer and the target RNA. Therefore, gapmers can be used to reduce the unwanted RNA level (i.e., gene knockdown) in a catalytic manner (Meng et al., 2015; Crooke, 2017). The DNA block in a gapmer is usually flanked (capped) by a short sequence of modified nucleotides to prevent exonuclease degradation.

“Masking” ASOs are commonly used as a steric block in the target RNA and, thereby, to modulate RNA splicing and suppress translation. The FDA has approved several ASOs acting through this mechanism to treat a variety of human diseases (Roberts et al., 2020; Tang et al., 2021a). For example, fomivirsen was the first FDA-approved ASO drug to treat cytomegalovirus (CMV) retinitis (approved in 1998; withdrawn in 2006 for lack of medical need) (Stein and Castanotto, 2017). Fomivirsen binds to the immediate early region 2 in the human CMV mRNA, halting the RNA translation of (IE)-2 protein which is crucial for viral replication (Geary et al., 2002). ASOs are also widely used for modulating RNA splicing in rare genetic diseases, such as Duchenne muscular dystrophy (DMD) and spinal muscular atrophy (SMA) (Tang et al., 2021b).

#### Chemical Modification in ASOs

Several chemical modifications of ASOs have been developed to improve their stability and cellular uptake (Crooke, 2017). For example, replacing the natural phosphodiester bridge with a phosphorothioate group in the ASO would significantly increase its half-life *in vivo* due to high serum protein binding and nuclease resistance (Temsamani et al., 1993). Phosphorothioate linkage in ASOs retains the RNase H1 recognition and is usually used throughout gapmers (Lulla et al., 2021). Alkylation of the 2’-OH in the ribose with a methoxyethyl group (MOE) in the ASO would enhance the hybridization stability and lessen the nonspecific binding (Dhuri et al., 2020). It is estimated that each MOE substitution increases the melting temperature (*T*_m_) by 2 °C (Freier and Altmann, 1997). Locked nucleic acid (LNA) is a class of modified ribose where the 2’-OH is linked to the 4’-CH *via* a constrained methylene bridge (Singh et al., 1998). The constrained LNA maintains a preferable conformation in RNA binding and, therefore, would significantly increase the hybridization stability in ASOs (+2–4°C in *T*_m_ per LNA substitution) (Koshkin et al., 1998). One or more LNAs can be used in ASOs, and the ASOs with interspersed combination of LNA and DNA nucleotides are also termed “mixmers” (Bernardo et al., 2012). A popular ASO form in clinical use is based on a phosphorodiamidate morpholino oligomer (PMO) skeleton. PMOs have morpholine subunits instead of ribose/deoxyribose and are linked by the phosphorodiamidate group (Dhuri et al., 2020). PMOs have various advantages, including reduced nonspecific binding imparted by the neutral charge and complete nuclease resistance (Dhuri et al., 2020).

#### Anti-SARS-CoV-2 ASOs

**FIGURE 5:**
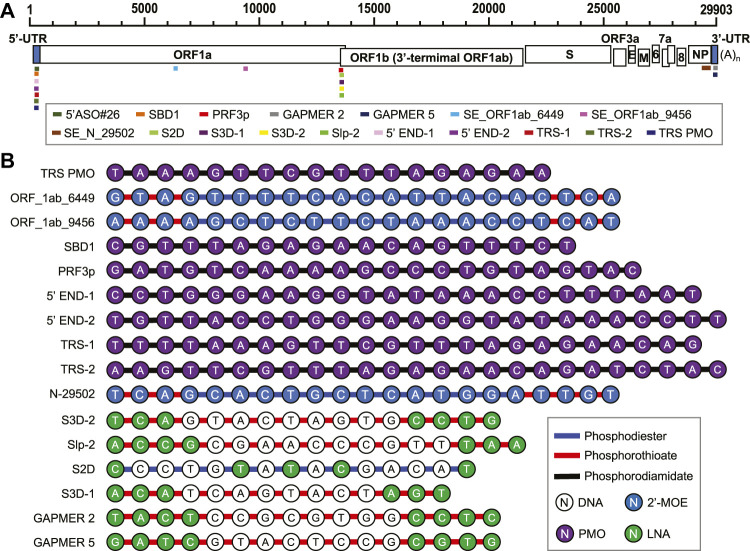
**(A)** Antiviral ASO binding sites in the SARS-CoV-2 genome. **(B)** Chemical composition of the anti-SARS-CoV-2 ASOs.

By using 3D antisense modeling, a PMO named PRF3p was optimized to target the Stem 3 region in the PFS element (Li et al., 2021b) (Figure 5). The PRF3p binding disrupted the pseudoknotted structure in the PFS element and inhibited the frameshift, eventually leading to a knockdown of the genes encoded by the ORF1b in the virus-infected 293T cells (Li et al., 2021b). Gapmers S2D, S3D-1, S2D-2, and Slp-2 targeting PFS elements were reported to have efficacy in Huh-7 inoculated with SARS-CoV-2 with a luciferase reporter (Zhang et al., 2021).

A PMO named SBD1 was designed to target the conserved TRS-L region in the SARS-CoV 5’ UTR (Figure 5), and thereby inhibited the “discontinuous” transcription (Li et al., 2021b). The suppression of sub-genomic RNA transcription ultimately led to the reduction of viral structural protein levels and virus titer (Li et al., 2021). Two PMOs, 5’END-1 and 5’END-2, targeted the viral 5’ UTR and were shown to inhibit the translation pre-initiation complex (Rosenke et al., 2021). The 5’-end of ORF1a is also a region for ASO-binding to have antiviral effects. Two 2’-MOE/phosphorothioate-modified ASOs targeting this region, SE_ORF1ab_6449 and SE_ORF1ab_9456, were reported to effectively inhibit SARS-CoV infection in Vero E6 cells (Figure 5) (Sun et al., 2021a). Gapmers 2 and 5 targeting the conserved S2M sequences in the 3’ UTR were demonstrated to have efficacy in degrading the viral RNA genome (Figure 5) (Lulla et al., 2021). The current development of ASO-based anti-SARS-CoV-2 agents is summarized in Table 1.

**TABLE 1:** ASO-based anti-SARS-CoV-2 agents that target the viral RNA genome.

Name	Chemistry^a^	Target gene	Target or ASO sequences	Length	References
5’ASO#26	Mixmer/PS	5’ UTR	29–44	16	Zhu et al. (2021)
SE_ORF1ab_6449	2’-MOE/PS	ORF1ab	6,451–6,471	21	Sun et al. (2021a)
SE_ORF1ab_9456	2’-MOE/PS	ORF1ab	9,458–9,478	21	Sun et al. (2021a)
SE_N_29502	2’-MOE/PS	ORF1ab	29,497–29,517	21	Sun et al. (2021a)
SBD1	PMO	5’UTR, TRS	59–72 & 79–85^b^	19	Li et al. (2021b)
PRF3p	PMO	PFS Element	13,503–13,506 and 13,534–13,551^b^	22	Li et al. (2021b)
GAPMER 2	Gapmer/PS	S2M, 3’UTR	29,734–29,749	16	Lulla et al. (2021)
GAPMER 5	Gapmer/PS	S2M, 3’UTR	29,739–29,754	16	Lulla et al. (2021)
S2D	Mixmer	PFS Element	13,526–13,540	15	Zhang et al. (2021)
S3D-1	Gapmer/PS	PFS Element	13,516-13,529	14	Zhang et al. (2021)
S3D-2	Gapmer/PS	PFS Element	13,511–13,526	16	Zhang et al. (2021)
Slp-2	Gapmer/PS	PFS element	13,463–13,479	17	Zhang et al. (2021)
5’END-1	PMO	5’UTR	1–24	24	Rosenke et al. (2021)
5’END-2	PMO	5’UTR	5–29	25	Rosenke et al. (2021)
TRS1	PMO	5’UTR, TRS	59–82	24	Rosenke et al. (2021)
TRS2	PMO	5’UTR, TRS	53–77	25	Rosenke et al. (2021)
TRS PMO	PMO	5’UTR, TRS	62–79	18	Li et al. (2021a)

a PS, phosphorothioate.

b 3D-ASOs.

### RNA-Degrading Chimeras

The RNA-degrading chimeras follow a well-established precedent from the protein field, namely, the proteolysis targeting chimera or PROTAC. PROTACs bind to their target protein using a guide arm as “bait”. The effector arm of PROTACs recruits an endogenous E3 ubiquitin ligase resulting in polyubiquitination and subsequent proteasomal degradation of the target protein (Schapira et al., 2019). The Disney group first extended this chimeric degrader concept to the RNA field by creating a ribonuclease targeting chimera (RIBOTAC) (Costales et al., 2018). RIBOTACs have been developed as a new class of chimeric molecules that use a guide arm to bind to the RNA sequence of interest. The effector arm of RIBOTAC would recruit the endogenous ribonuclease (RNase) L, causing degradation of the target RNA without affecting the host transcriptome (Costales et al., 2018; Disney, 2019; Costales et al., 2020; Liu et al., 2020; Meyer et al., 2020).

**FIGURE 6:**
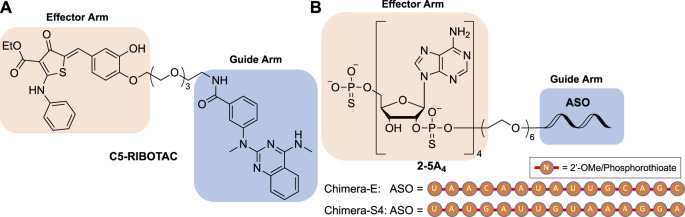
Structures of **(A)** small molecule-based and **(B)** ASO-based RIBOTACs targeting the SARS-CoV-2 RNA genome.

RNase L plays an essential role in an innate immune response pathway, namely the oligoadenylate synthetase (OAS)-RNase L pathway. In a viral infection, OAS senses dsRNA and synthesizes 2',5'-linked oligoadenylates (2–5A) that activate RNase L by dimerization (Naik et al., 1998). RNase L cleaves single-stranded (ss) RNA preferentially on UA, UG, and UU sites (Floyd-Smith et al., 1981; Wreschner et al., 1981), leading to global RNA degradation, arrest of protein synthesis, and apoptosis (Li et al., 2004). A small-molecule RNase L dimerizer (i.e., activator) was previously discovered (*K*_d_ = 18 µM to RNase L monomer), presenting a modest antiviral effect as a single agent against human parainfluenza virus in cells (Thakur et al., 2007). The structure of this RNase L dimerizer was further modified to serve as an RNase L recruiter fragment in RIBOTAC (Costales et al., 2018; Costales et al., 2020; Haniff et al., 2020; Liu et al., 2020). Recently, the Disney group discovered a series of compounds that bound to the attenuator hairpin in the PFS element and used them as the guide arm for the RIBOTAC modality (Haniff et al., 2020). One of the small-molecule RIBOTACs, C5-RIBOTAC, has been shown to reduce SARS-CoV-2 RNA levels in a cellular model (Figure 6A) (Haniff et al., 2020).

Following the first small-molecule RIBOTAC report, another type of nucleic acid-based RIBOTAC targeting SARS-CoV-2 demonstrating efficacy in virus-infected cells was also disclosed (Su et al., 2021). This type of RIBOTACs target the spike or envelope protein coding RNA using a 15-nucleotide complementary antisense oligonucleotide (ASO) as the guide arm and a 2',5'-linked tetraadenylate (2–5A_4_) as an RNase L recruiter (Figure 6B). These RIBOTACs have been shown to reduce viral titer in virus-infected Vero E6 cells (Su et al., 2021).

## Discussion

Molecules targeting conserved viral RNA sequences and structures are a newly emerged pharmacological modality that can significantly expand our antiviral arsenal. ASOs that recognize primary viral RNA sequences can be rapidly designed and optimized in early drug discovery. The major obstacles to the clinical use of ASOs are the unfavorable cellular uptake and distribution (Moschos et al., 2011; Geary et al., 2015). Recently, administration by inhalation has shown promising results in ASO delivery in lung tissues (Crosby et al., 2017; Berber et al., 2021), which will probably be useful for the treatment of respiratory viruses, such as SARS-CoV-2. Other technologies in ASO delivery have been advanced in the field, such as liposome-enclosed ASOs (Garbuzenko et al., 2010) and ASOs conjugated with cell-penetrating peptides (CPPs) (McClorey and Banerjee, 2018). These technologies have the potential to further improve the pharmacokinetics of ASOs as antivirals.

Targeting RNA structures will broaden the spectrum of the small-molecule “druggability”. Compared to traditional protein targets in viruses, such as RdRP and proteases, a completely different target specificity will be obtained for RNA ligands. As illustrated in the SARS-CoV-2 5’ UTR, the RNA structures but not the exact sequences are conserved across betacoronavirus strains (Yang and Leibowitz, 2015). Such structural conservation will likely make the structure-recognizing small molecules cross-active within the viral genus. Despite the above promising features, the *in vivo* activity and toxicity profile of RNA-targeting small molecules as antivirals are still obscure. Major efforts are required to address these issues before RNA-targeting molecules can be used as antiviral drugs in clinics.

## Conclusion

Fueled by the current advances in RNA-binding small molecules, ASOs, and RNA-degrading chimeras, RNA-targeting strategies have already been demonstrated the use in inhibiting SARS-CoV-2. With further advances in structure modeling for RNAs and understanding of the RNA-ligand interactions, the RNA-targeting drug discovery platforms have the potential to quickly generate antiviral candidates to address future viral outbreaks.

## References

[B1] Aldred K. J. Kerns R. J. Osheroff N. (2014). Mechanism of Quinolone Action and Resistance. Biochemistry 53, 1565–1574. 10.1021/bi5000564 24576155 PMC3985860

[B2] Baranov P. V. Henderson C. M. Anderson C. B. Gesteland R. F. Atkins J. F. Howard M. T. (2005). Programmed Ribosomal Frameshifting in Decoding the SARS-CoV Genome. Virology 332, 498–510. 10.1016/j.virol.2004.11.038 15680415 PMC7111862

[B3] Berber B. Aydin C. Kocabas F. Guney-Esken G. Yilancioglu K. Karadag-Alpaslan M. (2021). Gene Editing and RNAi Approaches for COVID-19 Diagnostics and Therapeutics. Gene Ther. 28, 290–305. 10.1038/s41434-020-00209-7 33318646 PMC7734466

[B4] Bernardo B. C. Gao X.-M. Winbanks C. E. Boey E. J. H. Tham Y. K. Kiriazis H. (2012). Therapeutic Inhibition of the miR-34 Family Attenuates Pathological Cardiac Remodeling and Improves Heart Function. Proc. Natl. Acad. Sci. 109, 17615–17620. 10.1073/pnas.1206432109 23047694 PMC3491509

[B5] Bhatt P. R. Scaiola A. Loughran G. Leibundgut M. Kratzel A. Meurs R. (2021). Structural Basis of Ribosomal Frameshifting during Translation of the SARS-CoV-2 RNA Genome. Science 372, 1306–1313. 10.1126/science.abf3546 34029205 PMC8168617

[B6] Ceraolo C. Giorgi F. M. (2020). Genomic Variance of the 2019‐nCoV Coronavirus. J. Med. Virol. 92, 522–528. 10.1002/jmv.25700 32027036 PMC7166773

[B7] Costales M. G. Aikawa H. Li Y. Childs-Disney J. L. Abegg D. Hoch D. G. (2020). Small-molecule Targeted Recruitment of a Nuclease to Cleave an Oncogenic RNA in a Mouse Model of Metastatic Cancer. Proc. Natl. Acad. Sci. USA 117, 2406–2411. 10.1073/pnas.1914286117 31964809 PMC7007575

[B8] Costales M. G. Matsumoto Y. Velagapudi S. P. Disney M. D. (2018). Small Molecule Targeted Recruitment of a Nuclease to RNA. J. Am. Chem. Soc. 140, 6741–6744. 10.1021/jacs.8b01233 29792692 PMC6100793

[B9] Crooke S. T. (2017). Molecular Mechanisms of Antisense Oligonucleotides. Nucleic Acid Ther. 27, 70–77. 10.1089/nat.2016.0656 28080221 PMC5372764

[B10] Crosby J. R. Zhao C. Jiang C. Bai D. Katz M. Greenlee S. (2017). Inhaled ENaC Antisense Oligonucleotide Ameliorates Cystic Fibrosis-like Lung Disease in Mice. J. Cystic Fibrosis 16, 671–680. 10.1016/j.jcf.2017.05.003 28539224

[B11] Dervan P. B. (1986). Design of Sequence-specific DNA-Binding Molecules. Science 232, 464–471. 10.1126/science.2421408 2421408

[B12] Dhuri K. Bechtold C. Quijano E. Pham H. Gupta A. Vikram A. (2020). Antisense Oligonucleotides: An Emerging Area in Drug Discovery and Development. J. Clin. Med. 9, 2004–1373. 10.3390/jcm9062004 PMC7355792 32604776

[B13] Disney M. D. (2019). Targeting RNA with Small Molecules to Capture Opportunities at the Intersection of Chemistry, Biology, and Medicine. J. Am. Chem. Soc. 141, 6776–6790. 10.1021/jacs.8b13419 30896935 PMC6541398

[B14] Escors D. Izeta A. Capiscol C. Enjuanes L. (2003). Transmissible Gastroenteritis Coronavirus Packaging Signal Is Located at the 5′ End of the Virus Genome. J. Virol. 77, 7890–7902. 10.1128/jvi.77.14.7890-7902.2003 12829829 PMC161917

[B15] Fedorova O. Jagdmann G. E. Adams R. L. Yuan L. Van Zandt M. C. Pyle A. M. (2018). Small Molecules that Target Group II Introns Are Potent Antifungal Agents. Nat. Chem. Biol. 14, 1073–1078. 10.1038/s41589-018-0142-0 30323219 PMC6239893

[B16] Fischer W. Eron J. J. Holman W. Cohen M. S. Fang L. Szewczyk L. J. (2021). Molnupiravir, an Oral Antiviral Treatment for COVID-19. medRxiv. 10.1101/2021.06.17.21258639

[B17] Floyd-Smith G. Slattery E. Lengyel P. (1981). Interferon Action: RNA Cleavage Pattern of a (2′-5′)Oligoadenylate-dependent Endonuclease. Science 212, 1030–1032. 10.1126/science.6165080 6165080

[B18] Freier S. Altmann K. H. (1997). The Ups and downs of Nucleic Acid Duplex Stability: Structure-Stability Studies on Chemically-Modified DNA:RNA Duplexes. Nucleic Acids Res. 25, 4429–4443. 10.1093/nar/25.22.4429 9358149 PMC147101

[B19] Fung T. S. Liu D. X. (2019). Human Coronavirus: Host-Pathogen Interaction. Annu. Rev. Microbiol. 73, 529–557. 10.1146/annurev-micro-020518-115759 31226023

[B20] Garbuzenko O. B. Saad M. Pozharov V. P. Reuhl K. R. Mainelis G. Minko T. (2010). Inhibition of Lung Tumor Growth by Complex Pulmonary Delivery of Drugs with Oligonucleotides as Suppressors of Cellular Resistance. Proc. Natl. Acad. Sci. 107, 10737–10742. 10.1073/pnas.1004604107 20498076 PMC2890783

[B21] Geary R. S. Henry S. P. Grillone L. R. (2002). Fomivirsen. Clin. Pharmacokinet. 41, 255–260. 10.2165/00003088-200241040-00002 11978144

[B22] Geary R. S. Norris D. Yu R. Bennett C. F. (2015). Pharmacokinetics, Biodistribution and Cell Uptake of Antisense Oligonucleotides. Adv. Drug Deliv. Rev. 87, 46–51. 10.1016/j.addr.2015.01.008 25666165

[B23] Goebel S. J. Hsue B. Dombrowski T. F. Masters P. S. (2004). Characterization of the RNA Components of a Putative Molecular Switch in the 3′ Untranslated Region of the Murine Coronavirus Genome. J. Virol. 78, 669–682. 10.1128/jvi.78.2.669-682.2004 14694098 PMC368785

[B24] Goebel S. J. Miller T. B. Bennett C. J. Bernard K. A. Masters P. S. (2007). A Hypervariable Region within the 3′ Cis -Acting Element of the Murine Coronavirus Genome Is Nonessential for RNA Synthesis but Affects Pathogenesis. J. Virol. 81, 1274–1287. 10.1128/JVI.00803-06 17093194 PMC1797510

[B25] Gottlieb R. L. Nirula A. Chen P. Boscia J. Heller B. Morris J. (2021). Effect of Bamlanivimab as Monotherapy or in Combination with Etesevimab on Viral Load in Patients with Mild to Moderate COVID-19. JAMA 325, 632–644. 10.1001/jama.2021.0202 33475701 PMC7821080

[B26] Hagedorn P. H. Pontoppidan M. Bisgaard T. S. Berrera M. Dieckmann A. Ebeling M. (2018). Identifying and Avoiding Off-Target Effects of RNase H-dependent Antisense Oligonucleotides in Mice. Nucleic Acids Res. 46, 5366–5380. 10.1093/nar/gky397 29790953 PMC6009603

[B27] Hagemeijer M. Rottier P. Haan C. (2012). Biogenesis and Dynamics of the Coronavirus Replicative Structures. Viruses 4, 3245–3269. 10.3390/v4113245 23202524 PMC3509692

[B28] Hamy F. Felder E. R. Heizmann G. Lazdins J. Aboul-ela F. Varani G. (1997). An Inhibitor of the Tat/TAR RNA Interaction that Effectively Suppresses HIV-1 Replication. Proc. Natl. Acad. Sci. 94, 3548–3553. 10.1073/pnas.94.8.3548 9108013 PMC20476

[B29] Haniff H. S. Tong Y. Liu X. Chen J. L. Suresh B. M. Andrews R. J. (2020). Targeting the SARS-CoV-2 RNA Genome with Small Molecule Binders and Ribonuclease Targeting Chimera (RIBOTAC) Degraders. ACS Cent. Sci. 6, 1713–1721. 10.1021/acscentsci.0c00984 33140033 PMC7553039

[B30] Harger J. W. Dinman J. D. (2003). An *In Vivo* Dual-Luciferase Assay System for Studying Translational Recoding in the Yeast *Saccharomyces cerevisiae* . RNA 9, 1019–1024. 10.1261/rna.5930803 12869712 PMC1236998

[B31] Hargrove A. E. (2020). Small Molecule-RNA Targeting: Starting with the Fundamentals. Chem. Commun. 56, 14744–14756. 10.1039/D0CC06796B PMC7845941 33201954

[B32] Hillen H. S. Kokic G. Farnung L. Dienemann C. Tegunov D. Cramer P. (2020). Structure of Replicating SARS-CoV-2 Polymerase. Nature 584, 154–156. 10.1038/s41586-020-2368-8 32438371

[B33] Huston N. C. Wan H. Strine M. S. de Cesaris Araujo Tavares R. Wilen C. B. Pyle A. M. (2021). Comprehensive *In Vivo* Secondary Structure of the SARS-CoV-2 Genome Reveals Novel Regulatory Motifs and Mechanisms. Mol. Cel 81, 584–598. e5. 10.1016/j.molcel.2020.12.041 PMC7775661 33444546

[B34] Jaklevic M. C. (2020). Oral Drug Approved for Spinal Muscular Atrophy. JAMA 324, 1026. 10.1001/jama.2020.16783 32930745

[B35] Kelly J. A. Olson A. N. Neupane K. Munshi S. San Emeterio J. Pollack L. (2020). Structural and Functional Conservation of the Programmed −1 Ribosomal Frameshift Signal of SARS Coronavirus 2 (SARS-CoV-2). J. Biol. Chem. 295, 10741–10748. 10.1074/jbc.AC120.013449 32571880 PMC7397099

[B36] Kim D. Lee J.-Y. Yang J.-S. Kim J. W. Kim V. N. Chang H. (2020). The Architecture of SARS-CoV-2 Transcriptome. Cell 181, 914–921. e10. 10.1016/j.cell.2020.04.011 32330414 PMC7179501

[B37] Knoops K. Kikkert M. Worm S. H. E. v. d. Zevenhoven-Dobbe J. C. van der Meer Y. Koster A. J. (2008). SARS-coronavirus Replication Is Supported by a Reticulovesicular Network of Modified Endoplasmic Reticulum. Plos Biol. 6, e226. 10.1371/journal.pbio.0060226 18798692 PMC2535663

[B38] Koshkin A. A. Singh S. K. Nielsen P. Rajwanshi V. K. Kumar R. Meldgaard M. (1998). LNA (Locked Nucleic Acids): Synthesis of the Adenine, Cytosine, Guanine, 5-methylcytosine, Thymine and Uracil Bicyclonucleoside Monomers, Oligomerisation, and Unprecedented Nucleic Acid Recognition. Tetrahedron 54, 3607–3630. 10.1016/S0040-4020(98)00094-5

[B39] Lan T. C. T. Allan M. F. Malsick L. E. Khandwala S. Nyeo S. S. Y. Sun Y. (2020). Insights into the Secondary Structural Ensembles of the Full SARS-CoV-2 RNA Genome in Infected Cells. bioRxiv. 10.1101/2020.06.29.178343 PMC8891300 35236847

[B40] Li C. Callahan A. J. Simon M. D. Totaro K. A. Mijalis A. J. Phadke K.-S. (2021a). Fully Automated Fast-Flow Synthesis of Antisense Phosphorodiamidate Morpholino Oligomers. Nat. Commun. 12, 4396. 10.1038/s41467-021-24598-4 34285203 PMC8292409

[B41] Li G. Xiang Y. Sabapathy K. Silverman R. H. (2004). An Apoptotic Signaling Pathway in the Interferon Antiviral Response Mediated by RNase L and C-Jun NH2-terminal Kinase. J. Biol. Chem. 279, 1123–1131. 10.1074/jbc.M305893200 14570908

[B42] Li Y. Garcia G. Arumugaswami V. Guo F. (2021b). Structure-based Design of Antisense Oligonucleotides that Inhibit SARS-CoV-2 Replication. bioRxiv. 10.1101/2021.08.23.457434

[B43] Liphardt J. Napthine S. Kontos H. Brierley I. (1999). Evidence for an RNA Pseudoknot Loop-helix Interaction Essential for Efficient −1 Ribosomal Frameshifting. J. Mol. Biol. 288, 321–335. 10.1006/jmbi.1999.2689 10329145 PMC7141562

[B44] Liu P. Yang D. Carter K. Masud F. Leibowitz J. L. (2013). Functional Analysis of the Stem Loop S3 and S4 Structures in the Coronavirus 3′UTR. Virology 443, 40–47. 10.1016/j.virol.2013.04.021 23683838 PMC3700632

[B45] Liu X. Haniff H. S. Childs-Disney J. L. Shuster A. Aikawa H. Adibekian A. (2020). Targeted Degradation of the Oncogenic MicroRNA 17-92 Cluster by Structure-Targeting Ligands. J. Am. Chem. Soc. 142, 6970–6982. 10.1021/jacs.9b13159 32233464 PMC7357852

[B46] Lulla V. Wandel M. P. Bandyra K. J. Ulferts R. Wu M. Dendooven T. (2021). Targeting the Conserved Stem Loop 2 Motif in the SARS-CoV-2 Genome. J. Virol. 95, e0066321. 10.1128/JVI.00663-21 33963053 PMC8223950

[B47] Ma X. R. Alugubelli Y. R. Ma Y. Vatansever E. C. Scott D. A. Qiao Y. (2021). MPI8 Is Potent against SARS‐CoV‐2 by Inhibiting Dually and Selectively the SARS‐CoV‐2 Main Protease and the Host Cathepsin L. ChemMedChem. 10.1002/cmdc.202100456 PMC8427127 34242492

[B48] Manfredonia I. Nithin C. Ponce-Salvatierra A. Ghosh P. Wirecki T. K. Marinus T. (2020). Genome-wide Mapping of SARS-CoV-2 RNA Structures Identifies Therapeutically-Relevant Elements. Nucleic Acids Res. 48, 12436–12452. 10.1093/nar/gkaa1053 33166999 PMC7736786

[B49] Manzanares-Meza L. D. Medina-Contreras O. (2020). SARS-CoV-2 and Influenza: a Comparative Overview and Treatment Implications. Boletín Médico Del. Hosp. Infantil de México 77, 262–273. 10.24875/BMHIM.20000183 33064680

[B50] Mathews D. H. Disney M. D. Childs J. L. Schroeder S. J. Zuker M. Turner D. H. (2004). Incorporating Chemical Modification Constraints into a Dynamic Programming Algorithm for Prediction of RNA Secondary Structure. Proc. Natl. Acad. Sci. 101, 7287–7292. 10.1073/pnas.0401799101 15123812 PMC409911

[B51] McClorey G. Banerjee S. (2018). Cell-Penetrating Peptides to Enhance Delivery of Oligonucleotide-Based Therapeutics. Biomedicines 6, 51. 10.3390/biomedicines6020051 PMC6027240 29734750

[B52] Meng L. Ward A. J. Chun S. Bennett C. F. Beaudet A. L. Rigo F. (2015). Towards a Therapy for Angelman Syndrome by Targeting a Long Non-coding RNA. Nature 518, 409–412. 10.1038/nature13975 25470045 PMC4351819

[B53] Meyer S. M. Williams C. C. Akahori Y. Tanaka T. Aikawa H. Tong Y. (2020). Small Molecule Recognition of Disease-Relevant RNA Structures. Chem. Soc. Rev. 49, 7167–7199. 10.1039/d0cs00560f 32975549 PMC7717589

[B54] Moschos S. A. Frick M. Taylor B. Turnpenny P. Graves H. Spink K. G. (2011). Uptake, Efficacy, and Systemic Distribution of Naked, Inhaled Short Interfering RNA (siRNA) and Locked Nucleic Acid (LNA) Antisense. Mol. Ther. 19, 2163–2168. 10.1038/mt.2011.206 21971426 PMC3242665

[B56] Naik S. Paranjape J. M. Silverman R. H. (1998). RNase L Dimerization in a Mammalian Two-Hybrid System in Response to 2',5'-oligoadenylates. Nucleic Acids Res. 26, 1522–1527. 10.1093/nar/26.6.1522 9490801 PMC147421

[B57] Owen D. R. Allerton C. M. N. Anderson A. S. Aschenbrenner L. Avery M. Berritt S. (2021). An Oral SARS-CoV-2 M Pro Inhibitor Clinical Candidate for the Treatment of COVID-19. Science, eabl4784. 10.1126/science.abl4784 34726479

[B58] Papargyri N. Pontoppidan M. Andersen M. R. Koch T. Hagedorn P. H. (2020). Chemical Diversity of Locked Nucleic Acid-Modified Antisense Oligonucleotides Allows Optimization of Pharmaceutical Properties. Mol. Ther. - Nucleic Acids 19, 706–717. 10.1016/j.omtn.2019.12.011 31951854 PMC6965521

[B59] Park S.-J. Kim Y.-G. Park H.-J. (2011). Identification of RNA Pseudoknot-Binding Ligand that Inhibits the −1 Ribosomal Frameshifting of SARS-Coronavirus by Structure-Based Virtual Screening. J. Am. Chem. Soc. 133, 10094–10100. 10.1021/ja1098325 21591761

[B60] Rangan R. Zheludev I. N. Hagey R. J. Pham E. A. Wayment-Steele H. K. Glenn J. S. (2020). RNA Genome Conservation and Secondary Structure in SARS-CoV-2 and SARS-Related Viruses: a First Look. RNA 26, 937–959. 10.1261/rna.076141.120 32398273 PMC7373990

[B61] Roberts T. C. Langer R. Wood M. J. A. (2020). Advances in Oligonucleotide Drug Delivery. Nat. Rev. Drug Discov. 19, 673–694. 10.1038/s41573-020-0075-7 32782413 PMC7419031

[B62] Robson F. Khan K. S. Le T. K. Paris C. Demirbag S. Barfuss P. (2020). Coronavirus RNA Proofreading: Molecular Basis and Therapeutic Targeting. Mol. Cel 79, 710–727. 10.1016/j.molcel.2020.07.027 PMC7402271 32853546

[B63] Roman C. Lewicka A. Koirala D. Li N.-S. Piccirilli J. A. (2021). The SARS-CoV-2 Programmed −1 Ribosomal Frameshifting Element Crystal Structure Solved to 2.09 Å Using Chaperone-Assisted RNA Crystallography. ACS Chem. Biol. 16, 1469–1481. 10.1021/acschembio.1c00324 34328734 PMC8353986

[B64] Rosenke K. Leventhal S. Moulton H. M. Hatlevig S. Hawman D. Feldmann H. (2021). Inhibition of SARS-CoV-2 in Vero Cell Cultures by Peptide-Conjugated Morpholino Oligomers. J. Antimicrob. Chemother. 76, 413–417. 10.1093/jac/dkaa460 33164048 PMC7717290

[B65] Roy S. Delling U. Chen C. H. Rosen C. A. Sonenberg N. (1990). A Bulge Structure in HIV-1 TAR RNA Is Required for Tat Binding and Tat-Mediated Trans-activation. Genes Dev. 4, 1365–1373. 10.1101/GAD.4.8.1365 2227414

[B66] Sanders W. Fritch E. J. Madden E. A. Graham R. L. Vincent H. A. Heise M. T. (2020). Comparative Analysis of Coronavirus Genomic RNA Structure Reveals Conservation in SARS-like Coronaviruses. bioRxiv. 10.1101/2020.06.15.153197

[B67] Sawicki S. G. Sawicki D. L. Siddell S. G. (2007). A Contemporary View of Coronavirus Transcription. J. Virol. 81, 20–29. 10.1128/JVI.01358-06 16928755 PMC1797243

[B68] Schapira M. Calabrese M. F. Bullock A. N. Crews C. M. (2019). Targeted Protein Degradation: Expanding the Toolbox. Nat. Rev. Drug Discov. 18, 949–963. 10.1038/s41573-019-0047-y 31666732

[B69] Schulze-Gahmen U. Hurley J. H. (2018). Structural Mechanism for HIV-1 TAR Loop Recognition by Tat and the Super Elongation Complex. Proc. Natl. Acad. Sci. USA 115, 12973–12978. 10.1073/pnas.1806438115 30514815 PMC6305006

[B70] Singh S. K. Koshkin A. a. Wengel J. Nielsen P. (1998). LNA (Locked Nucleic Acids): Synthesis and High-Affinity Nucleic Acid Recognition. Chem. Commun. 1998, 455–456. 10.1039/a708608c

[B71] Sola I. Almazán F. Zúñiga S. Enjuanes L. (2015). Continuous and Discontinuous RNA Synthesis in Coronaviruses. Annu. Rev. Virol. 2, 265–288. 10.1146/annurev-virology-100114-055218 26958916 PMC6025776

[B72] Stammler S. N. Cao S. Chen S.-J. Giedroc D. P. (2011). A Conserved RNA Pseudoknot in a Putative Molecular Switch Domain of the 3′-untranslated Region of Coronaviruses Is Only Marginally Stable. RNA 17, 1747–1759. 10.1261/rna.2816711 21799029 PMC3162339

[B73] Stein C. A. Castanotto D. (2017). FDA-approved Oligonucleotide Therapies in 2017. Mol. Ther. 25, 1069–1075. 10.1016/j.ymthe.2017.03.023 28366767 PMC5417833

[B74] Su X. Ma W. Feng D. Cheng B. Wang Q. Guo Z. (2021). Efficient Inhibition of SARS‐CoV‐2 Using Chimeric Antisense Oligonucleotides through RNase L Activation. Angew. Chem. Int. Ed. 60, 21662–21667. 10.1002/anie.202105942 PMC8426974 34278671

[B75] Sun L. Li P. Ju X. Rao J. Huang W. Ren L. (2021a). *In Vivo* structural Characterization of the SARS-CoV-2 RNA Genome Identifies Host Proteins Vulnerable to Repurposed Drugs. Cell 184, 1865–1883. e20. 10.1016/j.cell.2021.02.008 33636127 PMC7871767

[B76] Sun Y. Abriola L. Niederer R. O. Pedersen S. F. Alfajaro M. M. Silva Monteiro V. (2021b). Restriction of SARS-CoV-2 Replication by Targeting Programmed −1 Ribosomal Frameshifting. Proc. Natl. Acad. Sci. USA 118, e2023051118. 10.1073/pnas.2023051118 34185680 PMC8256030

[B77] Tang Z. Zhao J. Pearson Z. J. Boskovic Z. V. Wang J. (2021a). RNA-targeting Splicing Modifiers: Drug Development and Screening Assays. Molecules 26, 2263. 10.3390/molecules26082263 33919699 PMC8070285

[B78] Tang Z. Zhao J. Pearson Z. J. Boskovic Z. V. Wang J. (2021b). RNA-targeting Splicing Modifiers: Drug Development and Screening Assays. Molecules 26, 2263. 10.3390/molecules26082263 33919699 PMC8070285

[B79] Temsamani J. Tang J.-Y. Padmapriya A. Kubert M. Agrawal S. (1993). Pharmacokinetics, Biodistribution, and Stability of Capped Oligodeoxynucleotide Phosphorothioates in Mice. Antisense Res. Dev. 3, 277–284. 10.1089/ard.1993.3.277 8286928

[B80] Thakur C. S. Jha B. K. Dong B. Das Gupta J. Silverman K. M. Mao H. (2007). Small-molecule Activators of RNase L with Broad-Spectrum Antiviral Activity. Proc. Natl. Acad. Sci. 104, 9585–9590. 10.1073/pnas.0700590104 17535916 PMC1877983

[B81] Ursu A. Childs-Disney J. L. Andrews R. J. O’Leary C. A. Meyer S. M. Angelbello A. J. (2020). Design of Small Molecules Targeting RNA Structure from Sequence. Chem. Soc. Rev. 49, 7252–7270. 10.1039/d0cs00455c 32935689 PMC7707016

[B82] van Dorp L. Acman M. Richard D. Shaw L. P. Ford C. E. Ormond L. (2020). Emergence of Genomic Diversity and Recurrent Mutations in SARS-CoV-2. Infect. Genet. Evol. 83, 104351. 10.1016/j.meegid.2020.104351 32387564 PMC7199730

[B83] Van Egeren D. Novokhodko A. Stoddard M. Tran U. Zetter B. Rogers M. (2021). Risk of Rapid Evolutionary Escape from Biomedical Interventions Targeting SARS-CoV-2 Spike Protein. PLoS One 16, e0250780. 10.1371/journal.pone.0250780 33909660 PMC8081162

[B84] Vankadari N. Jeyasankar N. N. Lopes W. J. (2020). Structure of the SARS-CoV-2 Nsp1/5′-Untranslated Region Complex and Implications for Potential Therapeutic Targets, a Vaccine, and Virulence. J. Phys. Chem. Lett. 11, 9659–9668. 10.1021/acs.jpclett.0c02818 33135884

[B85] Vatansever E. C. Yang K. S. Drelich A. K. Kratch K. C. Cho C.-C. Kempaiah K. R. (2021). Bepridil Is Potent against SARS-CoV-2 *In Vitro* . Proc. Natl. Acad. Sci. USA 118, e2012201118. 10.1073/pnas.2012201118 33597253 PMC7958448

[B86] Wacker A. Weigand J. E. Akabayov S. R. Altincekic N. Bains J. K. Banijamali E. (2020). Secondary Structure Determination of Conserved SARS-CoV-2 RNA Elements by NMR Spectroscopy. Nucleic Acids Res. 48, 12415–12435. 10.1093/nar/gkaa1013 33167030 PMC7736788

[B87] Wang Y. Zhang D. Du G. Du R. Zhao J. Jin Y. (2020). Remdesivir in Adults with Severe COVID-19: a Randomised, Double-Blind, Placebo-Controlled, Multicentre Trial. The Lancet 395, 1569–1578. 10.1016/S0140-6736(20)31022-9 PMC7190303 32423584

[B88] Warner K. D. Hajdin C. E. Weeks K. M. (2018). Principles for Targeting RNA with Drug-like Small Molecules. Nat. Rev. Drug Discov. 17, 547–558. 10.1038/nrd.2018.93 29977051 PMC6420209

[B89] Wreschner D. H. McCauley J. W. Skehel J. J. Kerr I. M. (1981). Interferon Action-Sequence Specificity of the ppp(A2′p)nA-dependent Ribonuclease. Nature 289, 414–417. 10.1038/289414a0 6162102

[B90] Wu F. Zhao S. Yu B. Chen Y.-M. Wang W. Song Z.-G. (2020). A New Coronavirus Associated with Human Respiratory Disease in China. Nature 579, 265–269. 10.1038/s41586-020-2008-3 32015508 PMC7094943

[B91] Wu H.-Y. Brian D. A. (2010). Subgenomic Messenger RNA Amplification in Coronaviruses. Proc. Natl. Acad. Sci. 107, 12257–12262. 10.1073/pnas.1000378107 20562343 PMC2901459

[B92] Yang D. Leibowitz J. L. (2015). The Structure and Functions of Coronavirus Genomic 3′ and 5′ Ends. Virus. Res. 206, 120–133. 10.1016/j.virusres.2015.02.025 25736566 PMC4476908

[B93] Yoshida T. Naito Y. Yasuhara H. Sasaki K. Kawaji H. Kawai J. (2019). Evaluation of Off‐target Effects of Gapmer Antisense Oligonucleotides Using Human Cells. Genes Cells 24, 827–835. 10.1111/gtc.12730 31637814 PMC6915909

[B55] Zafferani M. Haddad C. Luo L. Davila-Calderon J. Yuan-Chiu L. (2021). Amilorides inhibit SARS-CoV-2 replication in vitro by targeting RNA structures. Sci. Adv. 7, eabl6096. 10.1126/sciadv.abl6096 PMC8626076 34826236

[B94] Zhang K. Zheludev I. N. Hagey R. J. Haslecker R. Hou Y. J. Kretsch R. (2021). Cryo-EM and Antisense Targeting of the 28-kDa Frameshift Stimulation Element from the SARS-CoV-2 RNA Genome. Nat. Struct. Mol. Biol. 28, 747–754. 10.1038/s41594-021-00653-y 34426697 PMC8848339

[B95] Zhao C. Qin G. Niu J. Wang Z. Wang C. Ren J. (2021). Targeting RNA G‐Quadruplex in SARS‐CoV‐2: A Promising Therapeutic Target for COVID‐19? Angew. Chem. Int. Ed. 60, 432–438. 10.1002/ANIE.202011419 32939952

[B96] Zhao J. Qiu J. Aryal S. Hackett J. Wang J. (2020). The RNA Architecture of the SARS-CoV-2 3′-Untranslated Region. Viruses 12, 1473. 10.3390/v12121473 PMC7766253 33371200

[B97] Zhu C. Lee J. Y. Woo J. Z. Xu L. Nguyenla X. Yamashiro L. H. (2021). An Intranasal ASO Therapeutic Targeting SARS-CoV-2. bioRxiv. 10.1101/2021.05.17.444397 PMC9349213 35922434

[B98] Ziv O. Price J. Shalamova L. Kamenova T. Goodfellow I. Weber F. (2020). The Short- and Long-Range RNA-RNA Interactome of SARS-CoV-2. Mol. Cel 80, 1067–1077. 10.1016/j.molcel.2020.11.004 PMC7643667 33259809

[B99] Zúñiga S. Sola I. Alonso S. Enjuanes L. (2004). Sequence Motifs Involved in the Regulation of Discontinuous Coronavirus Subgenomic RNA Synthesis. J. Virol. 78, 980–994. 10.1128/jvi.78.2.980-994.2004 14694129 PMC368802

[B100] Züst R. Miller T. B. Goebel S. J. Thiel V. Masters P. S. (2008). Genetic Interactions between an Essential 3′ Cis -Acting RNA Pseudoknot, Replicase Gene Products, and the Extreme 3′ End of the Mouse Coronavirus Genome. J. Virol. 82, 1214–1228. 10.1128/JVI.01690-07 18032506 PMC2224451

