# Corrigendum: Inhibition of SARS-CoV-2 by Targeting Conserved Viral RNA Structures and Sequences

**DOI:** 10.3389/fchem.2022.842171

**Published:** 2022-02-04

**Authors:** Shalakha Hegde, Zhichao Tang, Junxing Zhao, Jingxin Wang

**Affiliations:** Department of Medicinal Chemistry, University of Kansas, Lawrence, KS, United States

**Keywords:** SARS-CoV-2, antiviral, RNA-targeting, small molecule, antisense oligonucleotide, untranslated region, programmed frameshift, RIBOTAC

In the original article, there was a mistake in Figure 4 as published. Figure 4 was a duplication of **Figure 6**. The corrected Figure 4 appears below.

**FIGURE 4:**
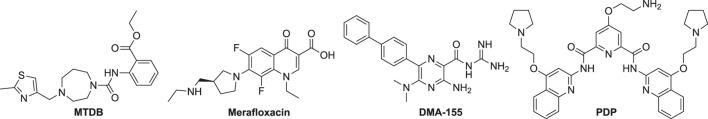
The small molecules with antiviral activities targeting the SARS-CoV-2 RNA genome.

The authors apologize for this error and state that this does not change the scientific conclusions of the article in any way. The original article has been updated.

